# The Effect of Sow Maternal Behavior on the Growth of Piglets and a Genome-Wide Association Study

**DOI:** 10.3390/ani13243753

**Published:** 2023-12-05

**Authors:** Xin Liu, Hanmei Li, Ligang Wang, Longchao Zhang, Lixian Wang

**Affiliations:** Institute of Animal Science, Chinese Academy of Agricultural Sciences, Beijing 100193, China; firstliuxin@163.com (X.L.); 18713593809@163.com (H.L.); ligwang@126.com (L.W.); zhlchias@163.com (L.Z.)

**Keywords:** maternal behavior, piglet growth, comparative analysis, GWAS

## Abstract

**Simple Summary:**

Sows’ maternal behavior is important for piglet survival and growth. It is critical to explore their genetic mechanisms for better breeding. Comparative analysis showed that piglets’ growth traits were strongly associated with sows’ maternal behaviors. Through a genome-wide association study (GWAS) for biting piglets (BP), crushing piglets (CP), trampling piglets (TP) and screaming test (ST) traits, several candidate genes and single nucleotide polymorphisms (SNPs) were found. These findings preliminarily elucidate the genetic basis of sows’ maternal behavior traits and provide markers for molecular breeding.

**Abstract:**

Sows’ maternal behavior is important for improving piglet survival and growth; thus, breeding for good mothering sows is necessary for pig production. However, there is little research on the genetic mechanism of maternal behavior. In this study, a comparative analysis of piglets’ growth traits between good and bad maternal behavior groups and a genome-wide association study (GWAS) was performed to elucidate the impact of sows’ maternal behavior on piglet growth and identify candidate genes and markers of sow’s maternal behaviors. Comparing the growth traits of piglets between good and bad sows’ maternal behavior groups, the results showed that the growth traits of piglets from sows with good maternal behavior were better than those from sows with bad maternal behavior and especially for the multiparous sows group, this comparative difference was significant. For the intensive study of the genetic mechanisms of sows’ maternal behavior, a total of 452 sows were genotyped using the Illumina Porcine 50K SNP Chip, and 4 traits, including biting piglets (BP), crushing piglets (CP), trampling piglets (TP) and screaming test (ST), were examined. Using a GWAS, 20 single nucleotide polymorphisms (SNPs) were found to be associated with these traits. Within 1 Mb upstream and downstream of the significant SNPs screened, 138 genes were obtained. After pathway enrichment and gene annotation, *HIP1*, *FZD9* and *HTR7* were identified as important candidate genes affecting sows’ maternal behaviors. These findings preliminarily elucidate the genetic basis of sows’ maternal behavior traits and provide candidate genes and markers for molecular breeding in pigs.

## 1. Introduction

For decades, pig breeding has mainly focused on lean meat growth rate, growth efficiency, a high feed conversion rate and a large litter size, and has made substantial genetic progress [1,2]. Yet, selection is likely to have caused a decrease in farrowing survival rates and a reduction in piglet maturity at birth [3]. The mortality of piglets before weaning has become an important issue in modern piglet production, affecting production efficiency and animal welfare [4]. Postnatal piglet deaths occur mainly due to starvation, crushing, hypothermia or their combinations and the survival of piglets depends largely on the care of their mothers in the first days after parturition [5]. Maternal behavior is important for improving piglet survival rates and breeding for good mothering sows is needed for production [6,7].

Maternal behaviors encompass food provision, guarding offspring and other species-specific maternal behavior expressions [8]. Maternal behavior establishes a relationship between parents and offspring through the exchange of biological signals. Sows’ behavior can be good or bad, and this directly affects the survival and growth of piglets. Sows exhibit some good maternal behaviors, such as nest building and sow communication, and also exhibit some poor maternal behaviors, such as biting piglets and pushing piglets. In order to obtain more benefits from the birth and survival of piglets and improve the maternal care and welfare of piglets during the lactation period, the selection for maternal behavior can be added to breeding goals [9]. Several years ago, the Nordic project was established to study sow maternal behavior with a focus on genetics, physiology and social environment [10]. To study the genetic mechanism of related traits, it is necessary to understand some basic genetic information about them. Some studies have been reported on the genetic parameter estimation of maternal behavior and its correlation with the survival and growth of piglets. Estimates of heritability of the maternal behavior traits during lactation ranged from 0.06 to 0.14 [11]. The heritability of the sows’ reaction to piglet screaming was found to be 0.16, according to the data recorded under field conditions and the estimated heritability for the scream test trait was 0.06 [9,10]. The heritability of maternal infanticide was estimated for daughter/sire (0.12–0.25) and daughter/dam (0.5–0.9) [12], showing significant additive genetic effects in primiparous sows [13]. These results showed that some maternal behavior traits were lowly to moderately heritable, indicating that genetic improvement for these traits would be slow. Genomic analysis methods offer a promising avenue for exploring key genes and genetic mechanisms to enhance the genetic improvement of specific traits. There have also been studies on the genetic correlation of maternal behavior in sows. For instance, in the study of genetic correlations between piglet survival and various behavioral traits, the scream test and avoidance traits showed significant correlations with piglet mortality [9]. Additionally, poor maternal ability was associated with more problems in terms of piglet crushing and savaging which could affect the survival of piglets [14]. So, there is evidence of a strong genetic correlation between the maternal behaviors of sows and piglet performance. Selection for maternal behavioral traits during production could indirectly contribute to improved piglet survival. Furthermore, investigations into the genetic mechanisms of maternal behavior have been conducted. Early studies utilized microsatellite markers to perform whole-genome linkage analyses on maternal infanticide in a White Duroc × Erhualian population, identifying candidate markers [15]. Subsequent studies in a White Duroc × Erhualian resource population detected quantitative trait loci (QTL) and associated genes for maternal infanticide behavior using single nucleotide polymorphisms (SNPs) and haplotypes. Notable QTL regions and candidate genes such as *ESR2*, *EAAT2* and *DRD1* were identified [13].

Although there have been numerous studies on the genetic parameters of maternal behaviors, due to the low heritabilities of some traits and unclear genetic mechanisms, it is difficult to conduct further genetic research and improvement on sow’s maternal behavior under past technologies. With the development of omics technology, it is advantageous to analyze the genetic mechanisms of complex traits and explore associated candidate genes. In this study, we conducted research on sows’ maternal behavior through a genome-wide association study (GWAS), in order to obtain significantly associated candidate genes and SNPs, which will provide theoretical support for future research on the genetic mechanism of sows’ maternal behaviors.

## 2. Materials and Methods

### 2.1. Ethics Statement

The study was approved by the Ethics Committee of the Institute of Animal Sciences of the Chinese Academy of Agricultural Sciences. All experimental protocols were conducted in accordance with the approved guidelines.

### 2.2. Animal Population and Phenotypic Data

The experimental population consisting of 452 Yorkshire sows was reared in identical intensive breeding conditions in Shanxi province, with the same feed and management. There were 125 gilts at the first farrowing (primiparous sows, PS) and 327 sows at the second or more farrowing (multiparous sow, MS).

Observation and recording of biting piglets (BP), crushing piglets (CP) and trampling piglets (TP) were carried out from birth to 24 h after giving birth by specially-trained observers positioned outside the pens to reduce interference with the natural behavior of sows. The piglet screaming test (ST) was carried out 72 h after parturition. The definitions of various maternal behaviors are listed in Table 1.

### 2.3. The Association between Sow Maternal Behavior and Piglet Growth Traits

The experimental population was divided into two groups for further analysis based on PS and MS. Then, in each group (PS or MS), the test sows were divided into two groups with good maternal behavior (GM) or bad maternal behavior (BM), based on the grouping rules. The grouping rules were mainly based on the occurrence of BP, CP and TP in sows and the intensity of the sow’s response in the ST test (details shown in Table 2). In order to understand the impact of sow maternal behavior on piglet growth, the piglets’ growth status of the GM and BM groups of the PS and MS sows were compared separately. The growth status indicators of piglets included weaning weight (WW), average weaning body weight (AWW) and weight gain of the weaning nest (WG) (WG = WW − birth weight). Differences in piglet growth traits between the GM and BM groups in PS and MS were separately analyzed using the T-test analysis method in SAS9.2 software (SAS INSTITUTE Inc., Cary, NC, USA).

### 2.4. Genotyping and Quality Control

Genomic DNA was isolated from ear tissue from 452 Yorkshire sows using the commercially available Q1Aamp DNA Mini Kit (QIAGEN, Hilden, Germany). The quantity and quality of extracted DNA were evaluated using a Nanodrop100 spectrophotometer and 1% agarose gel electrophoresis. All animals were genotyped using the GeneSeek Genomic Profiler (GGP) Porcine 50K chip (Illumina, San Diego, CA, USA), which contains 50,697 SNPs across the whole genome.

Quality control (QC) was performed using PLINK [16]. The QC criteria were both individual call rates and SNP call rates higher than 0.90, minor allele frequency (MAF) higher than 0.05, and Hardy–Weinberg equilibrium (HWE) *p* values less than 10^−6^. After filtering, a total of 452 pigs and 34,789 SNPs were retained for the subsequent study. 

### 2.5. Genome-Wide Association Study

Based on the phenotypic data, maternal behaviors were divided into two categories: counted traits (ST) and case–control traits (BP, CP and TP). Genome-wide complex trait analysis (GCTA) was utilized to perform GWAS analysis employing two different mixed models for different traits [17,18], and the specific description of models was as follows:

For case–control traits (BP, CP and TP), the model was

$$\mathrm{logit}\left(y\right)=\mu +X\beta +Z\gamma +u$$

For the counted trait (ST), the model was

y=μ+Xβ+Zγ+u+e

where y was the vector of the phenotypic values; μ was population mean; β was the vector of fixed effects and parity was added into the model as a fixed effect in this study, which was divided into primiparous sows and multiparous sows; γ was regression coefficient of substituting allele of tested SNP; u was the vector of polygenic effects; X was the incidence matrix of fixed effects; Z was the incidence matrix of SNP effects; and e was the vector of random residual error.

The false discovery rate (FDR) was implemented to determine the suggested threshold values [19] and was set to 0.01. The threshold P FDR value was set to FDR × n/m, in which n is the number of SNPs with *p* < 0.01, and m is the total number of SNPs. Manhattan and quantile–quantile (Q-Q) plots were drawn using the R package (http://cran.rproject.org/web/packages/gap/index.html) (accessed on 1 February 2023). 

### 2.6. Annotation of Candidate genes and Function Analysis

Gene annotation was performed according to genes’ physical positions using the BioMart tool in the Ensembl database (http://www.ensembl.org/) (accessed on 1 June 2023), based on the Sus scrofa 11.1 database. Candidate genes were retrieved by significant SNPs and extended 1 Mb up- and downstream from the significant SNPs. To further understand the function of potential candidate genes, genes were reviewed through the National Center for Biotechnology Information (NCBI: https://www.ncbi.nlm.nih.gov/) (accessed on 1 September 2023) and GeneCards (https://www.genecards.org/) (accessed on 1 September 2023), and OmicShare tools, a free online platform for data analysis (https://www.omicshare.com/tools) (accessed on 1 September 2023), was also used to perform pathway enrichment analysis, based on the KEGG pathway-related database.

## 3. Results

### 3.1. Description of Phenotypes and Comparative Analysis

A total of 452 Yorkshire sows were carefully observed and all maternal behaviors were scored. The frequency of sows’ maternal behaviors was calculated for both PS and MS and the specific description is as follows: the frequency of BP was 0.016 and 0.012, respectively; the frequency of CP was 0.480 and 0.394, respectively; the frequency of TP was 0.144 and 0.125, respectively; and the means of ST were 1.256 ± 1.33 and 0.917 ± 1.145, respectively. The results showed the frequency of maternal behaviors of PS was higher than that of MS sows.

The growth traits of the offspring were recorded and the descriptive statistics are shown in Table 3. The results showed that the piglets’ growth traits were strongly associated with sows’ maternal behaviors. The growth traits of piglets from sows with good maternal behavior were better than those from sows with bad maternal behavior, and especially for the offspring of MS, the differences in various traits reached significance (*p* < 0.05).

### 3.2. GWAS Results and Gene Annotation

**Figure 1:**
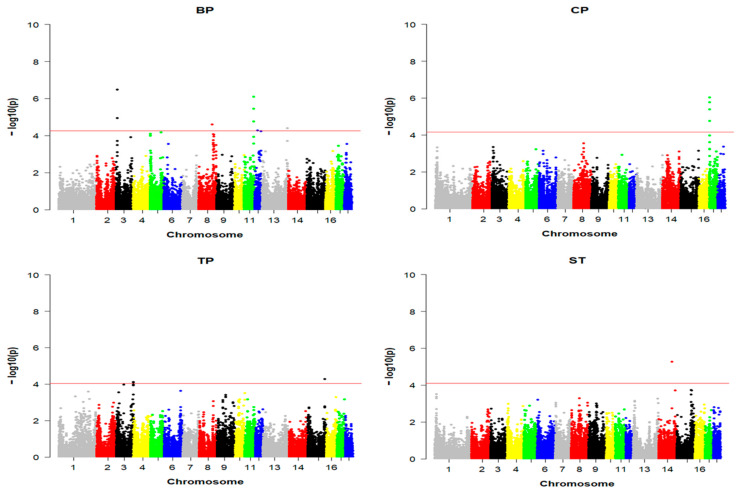
The Manhattan plots of four maternal behavior traits. The x-axis displays the distribution of chromosomes. The y-axis represents the −log10 (*p*-value) of each SNP in the GWAS analysis. The red line represents suggested significant threshold boundary and the significant SNPs related to each trait are located above the red line.

A total of 20 SNPs, which reached the suggestive significance level, were found to be associated with one of the maternal behavior traits (shown in Table 4). Manhattan plots were used to visualize the results for the association analysis between SNPs and traits (Figure 1). The significant SNPs of BP were concentrated on chromosomes (chr) 3 (containing four SNPs) and 11 (containing four SNPs), and there were three other significant SNPs located on chr8, chr12 and chr13. The most significant SNP among all was ALGA0112343, located in the intron of deltex E3 ubiquitin ligase 2 (*DTX2*) on chr3. The significance analysis associated with CP showed that all four significant SNPs were located on chr17, and the position was concentrated between 0.62 and 1.95 Mb. There were four significant SNPs associated with TP, among which the most significant one was located upstream of ArfGAP with FG repeats 1 (*AGFG1*) on chr15, and the other three were located at 13 Mb on chr3. Only one SNP was found to be significantly associated with ST in this study, and this SNP was located at 10 Mb on chr14, upstream of 5-hydroxytryptamine receptor 7 (*HTR7*). Potential genes were annotated within a 1 Mb range upstream and downstream of significant SNPs associated with maternal behavior traits, and 138 genes were obtained, including 96 genes for BP, seven genes for CP, 26 genes for TP and 9 genes for ST (details shown in Table S1). Through enrichment analysis of all genes, we found that they were enriched in some pathways related to neurodegenerative disease, which leads to cognitive, emotional and motor dysfunction. These pathways contained spinocerebellar ataxia, Huntington’s disease, Parkinson’s disease and pathways of neurodegeneration of multiple diseases (shown in Table S2), and some potential genes, including RNA polymerase II subunit J (*POLR2J*), huntingtin interacting protein 1 (*HIP1*), frizzled class receptor 9 (*FZD9*), ATPase sarcoplasmic/endoplasmic reticulum Ca^2+^ transporting 3 (*ATP2A3*), ubiquitin-conjugating enzyme E2 G1 (*UBE2G1*) and insulin receptor substrate 1 (*IRS1*), were enriched. After the annotation and function inquiry of these genes, we determined that *HIP1*, *FZD9* and *HTR7* might be important candidate genes affecting sows’ maternal behaviors.

## 4. Discussion

In the present study, the maternal behavior and piglet growth traits of Yorkshire sows were measured, and the GeneSeek GGP-Porcine 50K chip was used to genotype the sow population. Then, a comparative analysis of piglet’s growth traits between good and bad maternal behavior groups was performed and a GWAS between genotypes and four maternal behavior phenotypes was also carried out separately.

Pig production is dependent on the productivity of the sow. The breeding of sows for reproductive performance has increased litter size; however, the high mortality rate of piglets five days postpartum and even before weaning remains a problem faced in production. It has been reported that piglet deaths mainly occur within three days after delivery, with a mortality rate of 7–11%, and over half of piglet deaths are caused by factors such as hunger, sow crushing and trampling [20,21]. Additionally, a lack of maternal care in the early stages of birth may lead to abnormal neuroendocrine and immune regulation, thereby increasing the susceptibility of piglets to diseases [22]. Hence, the survival of piglets depends strongly on sows’ maternal care during the first days of life [23]. Unfortunately, in modern commercial pig production, the importance of maternal behavior is largely overlooked [24]. Sow maternal behavior is a major component of piglet survival and growth. Thus, consideration of improving sow maternal ability should be added to sow selection so as to increase overall production and welfare [25]. In this study, the sow maternal behaviors and related piglet growth traits were investigated from phenotypic data and genomics. Due to the differences in production performance and offspring capacity between PS and MS [26], analysis was performed separately for these two groups. The maternal behavior of Yorkshire sows, which were tested as the research object, was generally not very good due to long-term breeding for prolificacy and intensive management limitations, and the frequency of CP and TP was relatively high. Because of the differences existing in production performance and maternal care ability between PS and MS, the growth status of offspring between GM and BM was separately compared in PS and MS. The results showed that the growth traits of piglets from sows with good maternal behavior were better than those from sows with bad maternal behavior. This trend was observed in the PS sows, but the difference was not significant. This difference was particularly prominent in MS sows and was statistically significant, which may have been due to the better production performance and richer mothering ability of the MS sows. These results were consistent with Løvendahl’s research, which suggested that less aggressive sows were more responsive to the handling of their piglets [27]. Our results also demonstrate the importance of maternal behavior for the survival and growth of piglets.

Maternal behavior is easily overlooked in swine production, but increasing attention is being given to piglet survival rate, sow maternal behavior and animal welfare. Such traits are being incorporated into breeding objectives in the swine industry. Breeding sows for good maternal ability could offer an important and promising strategy for improving the postpartum survival rate of piglets [6,9]. However, due to the difficulty in measuring maternal behavior traits and their low heritability, there has not been much genetic progress in the past. Therefore, it is urgently needed to study the genetic mechanism of maternal behavior in sows, and candidate genes can be better identified taking advantage of the rapid development and widespread application of genomics. In this study, GWAS was performed for sow maternal behavior and some candidate genes were explored. We believed that maternal behavior was controlled by neural functions, such as emotional control, cognitive ability, motor disorders and so on. Therefore, the candidate genes for maternal behavior traits were identified, which were enriched in neurological diseases or related to neural regulation. *HIP1*, which was associated with BP, encodes membrane-associated protein binding to the huntingtin protein in the brain, and this interaction appears in Huntington’s disease, which causes symptoms including uncontrolled movement, emotional disturbances, psychiatric abnormalities, cognitive deficits and dementia [28]. The study of patients with intellectual disabilities, epilepsy and neurobehavioral problems found the deletion of HIP1 was sufficient to cause neurological disease and in subsequent mouse experiments, mice with a targeted mutation in the HIP1 gene showed neurodevelopmental disorders [29]. Another BP-associated candidate gene, *FZD9*, is located in the Williams syndrome (WS) deletion region; WS is a genetic neurodevelopmental disorder characterized by cognitive, behavioral, emotional and social symptoms, which is dependent on the genes involved in the deletion [30]. Research has shown that *FZD9*, as an important factor in neural cell regulation, is highly expressed in the hippocampus, and the deletion of the *FZD9* gene could affect the development of the nervous system and cause cognitive impairment by increasing the doubling and apoptosis of nerve cells [31,32]. *HTR7*, associated with ST, is considered an important candidate gene because it was a candidate locus in several neuropsychiatric disorders based on pharmacological studies [33]. *HTR7* genetic polymorphisms were found to have a relationship with schizophrenia according to GWAS [34]. One SNP in *HTR7* was demonstrated to be associated with the response to antidepressants in both bipolar and unipolar depression [35]. These three candidate genes have been confirmed to be related to neurological disorders and changes in their gene structure or function can lead to emotional loss, intellectual impairment, and motor disorders in humans or mice. Therefore, we speculate that these three candidate genes may also play an important genetic regulatory role in the performance of maternal behaviors in sows.

## 5. Conclusions

In this study, a comparative analysis of the growth traits of piglets from sows with good maternal behavior and bad maternal behavior was performed, and the results showed maternal behaviors and piglet growth traits were strongly related; specifically, the growth traits of piglets from sows with good maternal behavior were better than those of piglets from sows with bad maternal behavior and this difference reached the level of significance in multiparous sows. GWAS between genotypes and four maternal behavior phenotypes was also performed. Three candidate genes (*HIP1*, *FZD9* and *HTR7*) were identified following the analysis of significant results and gene annotation. These results will provide a foundation for the further study of phenotypes and genetic mechanisms of sows’ maternal behavior, and the explored genes and SNPs can be applied in selection for maternal behavior.

## Figures and Tables

**Table 1:** The description of sows’ maternal behaviors.

Traits	Description	Definition
BP	The piglet is bitten by sows or held in their mouths, even bitten to death	0: The piglet was not bitten; 1: The piglet was bitten.
CP	The piglet is trapped between the sow’s body and the floor	0: The piglet was not trapped; 1: The piglet was trapped.
TP	The piglet is trapped under the sow’s foot	0: The piglet was not trapped; 1: The piglet was trapped.
ST	Record the sound of the piglet screaming after being crushed. When the sow is lying down, the test operator plays a recording of its piglet’s screams to the sow, and then observers score the sow’s response to the piglet’s screams.	0: sow no response; 1: sow’s head movement; 2: sow’s body activity; 3: sow sitting; 4: sow standing; 5: sow contacting speaker.

**Table 2:** The description of grouping rules for GM and BM.

Sows	Group	Description
PS	GM1	BP, CP and TP did not occur and ST score ≥ 2
	BM1	Any one of BP, CP and TP occurred and ST score = 0
MS	GM2	BP, CP and TP did not occur and ST score ≥ 2
	BM2	Any one of BP, CP and TP occurred and ST score = 0

**Table 3:** Comparison of growth traits of piglets grouped with different maternal behaviors.

Sows	Group	Number of Sows	WW (kg) Mean ± Std err	AWW (kg) Mean ± Std err	WG (kg) Mean ± Std err
PS	GM1	15	65.17 ± 2.74	5.82 ± 0.19	47.12 ± 2.80
	BM1	23	61.05 ± 2.08	5.50 ± 0.16	46.27 ± 2.03
MS	GM2	42	64.41 ± 1.58 ^a^	5.85 ± 0.12 ^a^	47.807 ± 1.54 ^a^
	BM2	70	58.66 ± 1.67 ^b^	5.55 ± 0.09 ^b^	41.97 ± 1.70 ^b^

^a,b^ Means GM2 and BM2 with different superscripts indicate significant difference at *p* < 0.05. PS represents the primiparous sows, MS represents the multiparous sows, GM1 represents the PS sows with good maternal behavior, BM1 represents the PS sows with bad maternal behavior, GM2 represents the MS sows with good maternal behavior, BM2 represents the MS sows with bad maternal behavior, WW represents weaning weight, AWW represents average weaning body weight, WG represents weight gain of weaning nest.

**Table 4:** Significant SNPs associated with sows’ maternal behavior traits and their annotated candidate genes.

Trait	SNP	Chr	Position	*p* Value	Annotated Gene	Relationship between SNP and Annotation Gene Location
BP	ALGA0112343	3	9904449	3.24 × 10^−7^	*DTX2*	Intragenic
	WU_10.2_11_78734289	11	71480561	7.78 × 10^−7^	solute carrier family 10 member 2 (*SLC10A2*)	Downstream of gene
	ALGA0102815	11	71280437	3.60 × 10^−6^	ERCC excision repair 5, endonuclease (*ERCC5*)	Downstream of gene
	WU_10.2_11_78531862	11	71224492	3.60 × 10^−6^	*ERCC5*	Downstream of gene
	WU_10.2_3_9794925	3	9918901	1.13 × 10^−5^	*DTX2*	Intragenic
	WU_10.2_3_9822970	3	9956202	1.13 × 10^−5^	scavenger receptor cysteine-rich family member with 4 domains (*SSCD4*)	Upstream of gene
	3_9853222	3	9965529	1.13 × 10^−5^	*SSCD4*	Intragenic
	ALGA0063740	11	72023210	1.69 × 10^−5^	*SLC10A2*	Downstream of gene
	ALGA0049057	8	105770142	2.48 × 10^−5^	*N*-deacetylase and *N*-sulfotransferase 3 (*NDST3*)	Downstream of gene
	WU_10.2_13_208475892	13	198613309	4.01 × 10^−5^	RUNX family transcription factor 1 (*RUNX1*)	Intragenic
	H3GA0034642	12	49390321	5.18 × 10^−5^	spermatogenesis associated 22 (*SPATA22*)	Upstream of gene
CP	WU_10.2_17_1851007	17	1956407	9.08 × 10^−7^	sarcoglycan zeta (*SGCZ*)	Upstream of gene
	WU_10.2_17_1697296	17	1935638	1.64 × 10^−6^	*SGCZ*	Upstream of gene
	WU_10.2_17_1567203	17	1797169	4.11 × 10^−6^	DLC1 Rho GTPase activating protein (*DLC1*)	Downstream of gene
	WU_10.2_17_367361	17	620638	1.68 × 10^−5^	PEAK1-related, kinase-activating pseudokinase 1 (*PRAG1*)	Upstream of gene
TP	WU_10.2_15_142879832	15	128977934	5.17 × 10^−5^	*AGFG1*	Intragenic
	3_140838022	3	131693582	7.52 × 10^−5^	EARP complex and GARP complex interacting protein 1 (*EIPR1*)	Downstream of gene
	3_140608341	3	131398473	8.11 × 10^−5^	*EIPR1*	Intragenic
	3_140662560	3	131453400	8.11 × 10^−5^	*EIPR1*	Downstream of gene
ST	ALGA0080534	14	102414475	5.24 × 10^−6^	*HTR7*	Upstream of gene

Annotated gene: indicates the gene at or closest to the SNP.

## Data Availability

Genotypes are available in FigShare at: https://figshare.com/account/items/24709446/edit (accessed on 1 September 2023).

## Supplementary Materials

The following supporting information can be downloaded at: https://www.mdpi.com/article/10.3390/ani13243753/s1, Table S1: list of potential candidate genes. Table S2: list of candidate gene enrichment pathways. Figure S1: the Q-Q plots of four maternal behavior traits.
