# Supplementary material for: The Effect of Sow Maternal Behavior on the Growth of Piglets and a Genome-Wide Association Study
Source: Animals (Basel). 2023 Dec 5;13(24):3753. doi: 10.3390/ani13243753 (PMC10740555; doi:10.3390/ani13243753)
Supplement: Supplementary file 1 [file animals-13-03753-s001.zip › animals-2700943-supplementary.pdf]

Table S1 List of potential candidate genes

Trait	Chromosome	Candidate gene
Biting piglets (BP)	Chr3	*PLOD3*
		ZNHIT1
		CLDN15
		FIS1
		IFT22
		COL26A1
		MYL10
		CUX1
		SH2B2
		PRKRIP1
		ORAI2
		ALKBH4
		LRWD1
		POLR2J
		RASA4B
		UPK3BL2
		UPK3B
		DTX2
		ZP3
		SSC4D
		YWHAG
		HSPB1
		SRRM3
		MDH2
		STYXL1
		TMEM120A
		POR
		RHBDD2
		CCL24
		CCL26
		HIP1
		POM121C
		NSUN5
		TRIM50
		FKBP6
		FZD9
		BAZ1B
		TBL2
		BCL7B
		MLXIPL
		VPS37D
		DNAJC30

		BUD23
	Chr11	*TPP2*
		METTL21C
		CCDC168
		TEX30
		POGLUT2
		BIVM
		ERCC5
		SLC10A2
	Chr8	*SYNPO2*
		SEC24D
		METTL14
		PRSS12
		NDST3
		TRAM1L1
	Chr13	*SLC5A3*
		MRPS6
		KCNE2
		SMIM11
		C13H21orf140
		SMIM34
		KCNE1
		RCAN1
		CLIC6
		RUNX1
		SETD4
	Chr12	*SRR*
		TSR1
		SGSM2
		MNT
		METTL16
		PAFAH1B1
		CLUH
		RAP1GAP2
		SPATA22
		ASPA
		TRPV3
		TRPV1
		SHPK
		CTNS
		TAX1BP3
		EMC6
		P2RX5
		ITGAE

		HASPIN
		NCBP3
		CAMKK1
		P2RX1
		ATP2A3
		ZZEF1
		CYB5D2
		ANKFY1
		UBE2G1
		SPNS3
Crushing piglets (CP)	Chr17	*CLDN23*
		PRAG1
		TRMT9B
		DLC1
		C17H8orf48
		SGCZ
		TRNAE-UUC
Trampling piglets (TP)	Chr15	*IRS1*
		RHBDD1
		COL4A4
		COL4A3
		MFF
		TM4SF20
		AGFG1
		SLC19A3
		CCL20
		DAW1
		SPHKAP
	Chr3	*DCDC2C*
		ALLC
		COLEC11
		RPS7
		RNASEH1
		ADI1
		TRAPPC12
		EIPR1
		MYT1L
		PXDN
		TPO
		TMEM18
		ALKAL2
		ACP1
		SH3YL1
Piglets screaming test (ST)	Chr14	*PANK1*

			KIF20B
			HTR7
			RPP30
			ANKRD1
			PCGF5
			HECTD2
			PPP1R3C
			TNKS2

Table S2: List of candidate genes enrichment neurodegeneration pathways.

**KEGG_class**	**Pathway**	**Pathway_ID**	**Genes**
Signaling molecules and interaction	Neuroactive ligand-receptor interaction	ko04080	*P2RX5, P2RX1, HTR7, TRPV1*
Neurodegenerative disease	Pathways of neurodegeneration - multiple diseases	ko05022	*ATP2A3, HIP1, UBE2G1, FZD9*
Neurodegenerative disease	Alzheimer disease	ko05010	*ATP2A3, IRS1, FZD9*
Neurodegenerative disease	Spinocerebellar ataxia	ko05017	*ATP2A3*
Neurodegenerative disease	Huntington disease	ko05016	*POLR2J, HIP1*
Neurodegenerative disease	Parkinson disease	ko05012	*UBE2G1*
Neurodegenerative disease	Amyotrophic lateral sclerosis	ko05014	*POM121C*
Nervous system	Neurotrophin signaling pathway	ko04722	*SH2B2, IRS1*

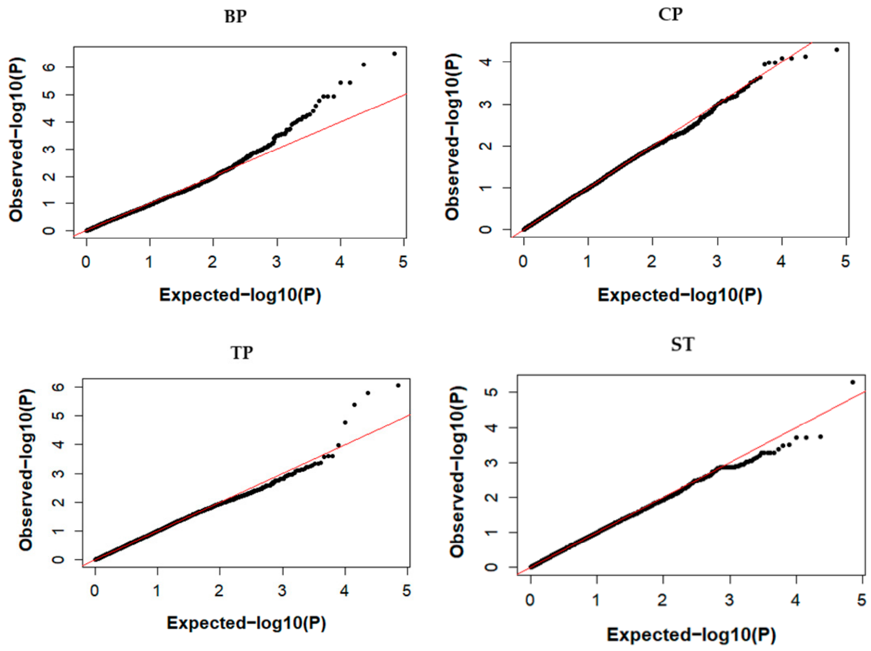

Figure S1. The Q-Q plots of four maternal behavior traits.
